# Assessing prostate, bladder and rectal doses during image guided radiation therapy — need for plan adaptation?

**DOI:** 10.1120/jacmp.v10i3.2883

**Published:** 2009-07-09

**Authors:** Raj Varadhan, Susanta K Hui, Sarah Way, Kurt Nisi

**Affiliations:** ^1^ Minneapolis Radiation Oncology North Radiation Therapy Center Robbinsdale MN U.S.A.; ^2^ Department of Therapeutic Radiology University of Minnesota Minneapolis MN U.S.A.

**Keywords:** adaptive radiotherapy, plan adaptation, image‐guided radiotherapy

## Abstract

The primary application of Image‐Guided Radiotherapy (IGRT) in the treatment of localized prostate cancer has been to assist precise dose delivery to the tumor. With the ability to use in‐room Computed Tomography (CT) imaging modalities, the prostate, bladder and rectum can be imaged before each treatment and the actual doses delivered to these organs can be tracked using anatomy of the day. This study evaluates the dosimetric uncertainties caused by interfraction organ variation during IGRT for 10 patients using kilovoltage cone beam CT (kvCBCT) on the Elekta Synergy system and megavoltage CT (MVCT) on the TomoTherapy Hi·Art System. The actual delivered doses to the prostate, bladder and rectum were based on dose recomputation using CT anatomy of the day. The feasibility of dose calculation accuracy in kvCBCT images from the Elekta Synergy system was investigated using the ComTom phantom. Additionally, low contrast resolution, image uniformity, and spatial resolution between the three imaging modalities of kilovoltage CT (kvCT), kvCBCT and MVCT images, were quantitatively evaluated using the Catphan 600 phantom. The Planned Adaptive software was used on the TomoTherapy Hi·Art system to construct a cumulative Dose Volume Histogram (DVH), incorporating anatomical information provided by the daily MVCT scans. The cumulative DVH was examined to identify large deviation (10% or greater) between the planned and delivered mean doses. The study proposes a framework that applies the cumulative DVH to evaluate and adapt plans that are based on actual delivered doses. Due to the large deviation in CT number (›300 HU) between the kvCBCT images and the kvCT, a direct dose recomputation on the kvCBCT images from the Elekta Synergy system was found to be inaccurate. The maximum deviation to the prostate was only 2.7% in our kvCBCT study, when compared to the daily prescribed dose. However, there was a large daily variation in rectum and bladder doses based on the anatomy of the day. The maximum variation in rectum and bladder volumes receiving the percentage of prescribed dose was 12% and 40%, respectively. We have shown that by using Planned Adaptive software on the TomoTherapy Hi·Art system, plans can be adapted based on the image feedback from daily MVCT scans to allow the actual delivered doses to closely track the original planned doses.

PACS number: 87.53.Tf

## I. INTRODUCTION

The goal of a radiation treatment is to ensure that the target receives accurate and adequate dose coverage, while the dose to the critical structures is kept as low as possible. Intensity‐Modulated Radiation Therapy (IMRT)^(^ ^1^ ^–^ ^3^ ^)^ and IGRT have led to more precise conformal radiation therapy. Conformal therapy has the potential to enhance the therapeutic ratio (dose to tumor/organ at risk (OAR)). However, due to the complexity of treatment delivery and variation in patient/tumor intrafraction and interfraction position, treatment may still pose risks for a geographic miss.^(^ ^4^ ^,^ ^5^ ^)^

The use of CT imaging in IGRT technology to localize the prostate, bladder, and rectum each day has made it possible to deliver the dose to the prostate more precisely. It is well known that the confirmation of the relative position and shape of the target and organs at risk during daily fractionated treatment is of fundamental importance to accurate dose delivery.^(6)^ Although the primary aim of IGRT technologies in the treatment of prostate cancer is to accurately localize the tumor for precise targeting, these technologies are also capable of monitoring changes in the filling and shape of the bladder and rectum. The ability to monitor and quantify the daily changes in these critical structures is necessary to track the actual dose delivered to them.

The current study evaluates 10 patients for the dosimetric changes resulting from interfraction organ motion associated with the treatment of prostate cancer. The two IGRT technologies used in the study include megavoltage CT (MVCT) localization on the TomoTherapy Hi·Art machine and kilovoltage cone‐beam CT (kvCBCT) localization using the Elekta Synergy system. A framework that can be applied to adapt plans for patients treated on the TomoTherapy Hi‐Art system was created. The framework includes a method to analyze the cumulative Dose Volume Histogram (DVH) calculated by the Planned Adaptive software. These evaluations can then be incorporated into a plan modification with the aim of minimizing the differences between planned and delivered doses.

Although previous studies^(7)^ with the tomotherapy system have demonstrated daily dose recalculations, to the best of our knowledge our study is the first one to attempt to create a summation dose and evaluate the dosimetric impact of taking into account the changes in daily parameters. The dosimetric information can be used to modify a patient plan or planning target volume (PTV) margins based on the evaluation of actual dose received.

## II. MATERIALS AND METHODS

### A. kvCBCT dose calculation accuracy on Elekta Synergy system

It is well known that compared to kvCT images, CBCT images suffer from an increased contribution of scatter.^(8)^ In general, X‐ray scatter reduces image contrast, increases image noise, and may introduce reconstruction error into CBCT images. In addition, the contribution of scatter is patient geometry dependent. Consequently the CT numbers of a CBCT scan cannot be simply converted to electron density and directly used for dose recomputation, as this may lead to dose errors.^(^ ^9^ ^,^ ^10^ ^)^ The usability of CBCT datasets for dose calculations has been investigated in the literature primarily for the Varian On‐Board Imaging (OBI) system. The investigations in the Varian OBI system showed only small differences in density calibration (less than 10 CT number) between kvCT and kvCBCT images using the Catphan600 phantom which makes it easily available for treatment planning.^(^ ^9^ ^–^ ^11^ ^)^ In contrast, the kvCBCT images from the Elekta Synergy system have shown large deviations in CT number and, as a result, an elaborate correction strategy is required.^(12)^ Recently it was shown that, even for the Catphan600 phantom, the CT numbers on the kvCBCT images from Elekta Synergy system were highly variable (depending on the image acquisition parameters) when compared with kvCT scans.^(13)^

**Figure 1:**
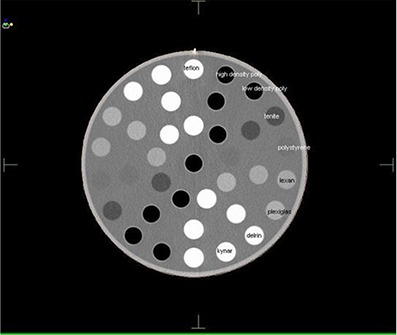
kvCT image of the ComTom Phantom.

The feasibility of direct dose recomputation on the kvCBCT images was investigated in this study using the ComTom CT Phantom. The ComTom phantom consists of 37 pins, each 1 inch in diameter, which are arranged in 3 concentric rings. There are 18 pins in the outer ring, each spaced 20 degrees apart. There are 9 pins in the middle and inner rings, respectively, all spaced 40 degrees apart. The CT numbers of the 9 pins plus air encompass the range of X‐ray attenuation normally found for human tissue. The relative electron density of the materials in this phantom (compared to a value of 1.0 for water) varied from 1.87 in Tefon to 0.15 for low‐density polyurethane. The kvCT scan of the phantom is shown in Fig. 1.

### B. kvCBCT patient study on Elekta Synergy system

The actual dose delivered to the prostate, bladder, and rectum for 5 patients was investigated by using the daily anatomy information provided by kvCBCT images from the Elekta Synergy system. Our standard IMRT treatment for definitive prostate cancer includes 7 equally spaced beams using 10 MV photons in a step‐and‐shoot delivery. All patients were treated to a dose of 75.6 Gy in 42 fractions. The treatments were planned and optimized using the CMS XiO treatment planning system and the dose calculation was performed using a convolution/superposition algorithm. The PTV margin routinely used at our institution for prostate IMRT is 1 cm in the superior/inferior direction and 8 mm everywhere else, except posteriorly – where the margin is 5 mm. The patients were instructed to have a “full bladder” at the time of CT simulation and during daily treatment. The current study did not include any analysis of seminal vesicles coverage.

One full volumetric kvCBCT study set was randomly chosen for every patient from each week of treatment. A total of 9 CT study sets (Week 1 to Week 9) were used for each patient to analyze the prostate, bladder, and rectal volume changes and their impact on dosimetry. The kvCBCT scans were manually contoured by the same radiation oncologist to account for any deformation in the target, rectum, and bladder. The kvCBCT scans were fused with the treatment planning CT scans and the dose was recomputed on the treatment planning CT (kvCT) scan. A two‐step process was used in registering the kvCT (primary study set) with the kvCBCT (secondary studyset) images. The primary and secondary studysets were transferred to the CMS Focal workstation. An automatic registration based on soft tissue match was performed to automate the alignment between the two studysets. The software computes the geometric transformation that best registers corresponding anatomic details in the two studysets of the same patient's anatomy. The alignment criterion is mutual information (MI), which is a measure of the statistical similarity of the overlapping data. The transformation that gives the maximum value of MI is considered to be the best registration. In the second step, interactive registration was used to further refine the automatic registration performed by the software. The same radiation oncologist manually inspected and refined the alignment of the prostate between the kvCBCT and kvCT studysets based on soft tissue match.

### C. Soft tissue contrast comparison of kvCT, kvCBCT, and MVCT scans

The Catphan600 phantom (Phantom Laboratory, Salem, NY) was used in order to quantitatively evaluate and compare low contrast resolution, image uniformity, and spatial resolution between the three imaging modalities (kvCT, kvCBCT, MVCT). The kvCT, kvCBCT, and MVCT images of CTP 404 module of the Catphan600 phantom are given below in Figs. 2(a)–2(c).

**Figure 2(a):**
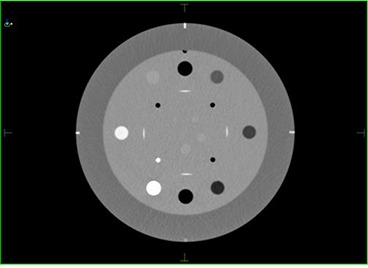
kvCT image of the CTP 404 module of Catphan Phantom.

**Figure 2(b):**
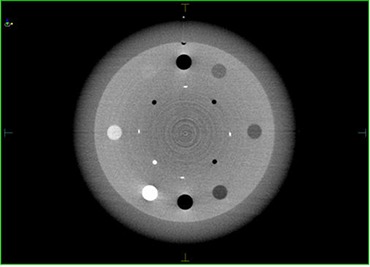
kvCBCT image of the CTP 404 module of Catphan Phantom.

**Figure 2(c):**
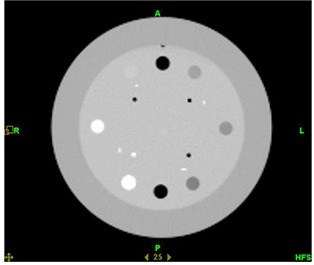
MVCT image of the CTP 404 module of Catphan Phantom.

Low contrast resolution (LCR) is the ability of the imaging system to distinguish between relatively large objects which differ only slightly in density from uniform background.^(14)^

The 3D low contrast resolution is computed from the mean and standard deviation of the pixel values of polystyrene and LDPE found in the CTP 404 module of the Catphan phantom using the formula:^(^ ^15^ ^,^ ^16^ ^)^

(1) $$LCR=\frac{2.75*(SD_{\text{poly}}+SD_{\text{LDPE}})}{\left({\text{Mean}}_{\text{Poly}}-{\text{Mean}}_{\text{LDPE}}\right)}$$

where we have assumed

(2) $$5.5=\frac{\left(No\min alCTnumberPoly-No\min alCTnumberLDPE\right)}{10}$$

Image artifacts due to equipment design, beam hardening or image reconstruction software can manifest themselves as systematic CT number variations. Hence, scanning a uniform phantom and sampling CT numbers in the fixed areas can quantify the presence of systematic variations. The 3D uniformity is computed from the pixel values of three locations in the CTP 486 uniformity module of the Catphan600 phantom using the formula.^(^ ^15^ ^,^ ^16^ ^)^

(3) $$3D\;Uniformity=\frac{Mean(high)-Mean(low)}{Mean(high)}*100$$

Spatial resolution characterizes the imaging system's ability to distinguish between two very small objects placed closely together. Spatial resolution measurements are performed with objects that have high contrast from uniform background. Spatial resolution is frequently referred to as high contrast resolution.^(17)^ The 3D high‐contrast or spatial resolution of the three imaging modalities was calculated by imaging and measuring the resolution pattern on the Catphan600 phantom (CTP 528 module).

### D. Adaptive tomotherapy

The details of MVCT image reconstruction during tomotherapy are well known and have been discussed by Ruchala et al.^(18)^ The energy of the MVCT beam (3.5 MV) is lower than that of the treatment beam (6 MV). The accuracy of dose calculation on the MVCT images was reported by Langen et al.,^(19)^ and has been independently verified at our institution as well.

The MVCT images are limited to a 40 cm circle of reconstruction due to the limitation of the maximum tomotherapy collimator width; whereas kvCT studies usually have a 50 cm circle of reconstruction, or larger in case of big bore CT scanners. MVCT scans are also typically shorter in the patient's craniocaudal direction to save time and reduce the imaging dose. Planned Adaptive software inserts the 40 cm field of view MVCT images into the corresponding kvCT treatment planning study by creating a combined MVCT/kvCT image studyset. This is referred to as the merged image. Typically kvCT images are acquired with a slice thickness of 3 mm and MVCT scanning on tomotherapy has three possible slice thicknesses: fine (2 mm), normal (4 mm) and coarse (6 mm). Therefore, interpolation within the MVCT image set is required to maintain a uniform 3 mm slice thickness. A different image‐value density table (IVDT) is used for performing dose calculations with MVCT images due to the higher beam energy of the tomotherapy unit (3.5 MV) as compared to the kvCT images. It has already been shown that the dose calculation is accurate using the merged images on the Planned Adaptive software when compared to the same plan using the kvCT image.^(20)^

In the Planned Adaptive software, the original contours used for treatment planning on the kvCT studyset are overlaid on the merged images, and they are recontoured, if necessary, based on anatomy of the day. Using the merged images as the imaging dataset for adaptive plans assumes that the regions of interest outside the MVCT scan in patient anatomy have not significantly changed because the MVCT images cover only limited length in the craniocaudal direction. Planned Adaptive software calculates verification doses for each patient. This is done by applying the daily delivery sinogram (based on the original kvCT plan) in the calculation of dose distribution on the merged image.

Through the Planned Adaptive software, a summation dose, which is the addition of verification doses from each treatment fraction, was generated and compared against the planned dose. Once the summation doses have been created, a cumulative DVH is constructed in the Planned Adaptive software. Planned Adaptive software facilitates the modification of structures (based on patterns of accumulated dose that may have resulted in over‐ or underdosage) in the merged image set. The resultant modified structures are then transferred to the Tomotherapy Planning Station for optimization of an “adaptive” plan. Adaptive planning allows adjustment of the remaining treatments to correct for changes that have occurred up to that point in treatment. Depending upon the anatomical site and clinical scenario, additional verifications and adaptive plans can be generated to correct for further anatomy variations. This paradigm is called Adaptive Tomotherapy Planning.

Five prostate patients were randomly chosen for this part of the study. All patients were treated in supine position using the Helical Tomotherapy unit at University of Minnesota. The patients were implanted with three gold seed markers to help align the MVCT studyset with the kvCT studyset and also to minimize inter‐user variability in registering images.

The positional variations of interfraction organ motion for each treatment fraction were systematically monitored and characterized using onboard MVCT images. The registration values used to position the patient at the time of treatment were used to correct the MVCT scan when creating the merged scan. The rectum and bladder were recontoured manually on merged studysets incorporating the bladder and rectal daily variation as determined on the MVCT scan. There were only minimal changes in the prostate target volume definition on the MVCT scans as compared to kvCT scan. The merged images created with the MVCT scans were then used to create adaptive treatment plans using the Tomotherapy Planning Station.

The reconstructed doses were compared with calculated treatment planning doses for individual organs through cumulative dose volume histograms (DVHs). The purpose of the comparison was to determine if treatment plan improvements can be dosimetrically significant, and to distinguish between clinically significant and insignificant anatomy changes. Cumulative DVHs from the Planned Adaptive software were analyzed for each patient and adaptive radiotherapy strategies were formed based on our analysis of these 5 patients.

## III. RESULTS

### A. kvCBCT dose calculation accuracy on Elekta Synergy system

**Figure 3:**
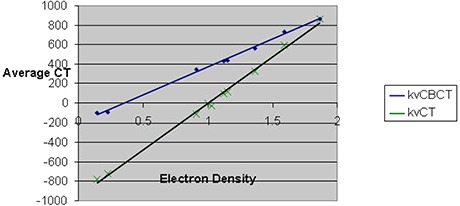
Relative electron density vs. CT number variation for kvCBCT and kvCT scans of ComTom Phantom.

The CT number derived from the kvCBCT image was found to vary considerably (average variation of 283 HU) from the kvCT image as seen in Fig. 3. The CT numbers derived from the kvCBCT scans showed the largest deviation from the corresponding kvCT image values for low relative electron density materials such as polyurethane, with a maximum deviation of 684 HU for the low density polyurethane. Further, the CT number reproducibility for the same material 1 cm superior and inferior to a given central axis slice varied by as much as ±200HU compared to the value on the central axis slice. Hence, it was concluded that direct dose recomputation on Elekta kvCBCT scans is not accurate or feasible at this time.

### B. kvCBCT patient study on Elekta Synergy system

Before each treatment, a kvCBCT scan was acquired and the prostate was aligned to the kvCT. The same physician was present and performed the alignment of kvCBCT with kvCT to eliminate inter‐user variability and interpretation of soft tissue images. The CBCT scan for the prostate was imaged at 120 kVp and 1040 mAs. Based on our measurements on the CIRS body phantom, this is equivalent to an imaging dose of 2.8 cGy at the center per day, for a total of 118 cGy over 42 fractions. This dose was not added to the actual treatment dose in dose comparisons.

**Figure 4:**
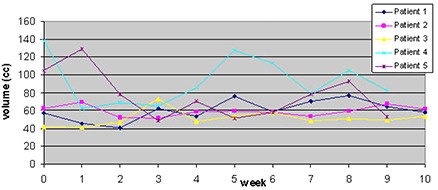
Changes in rectal volume over the course of treatment (42 fractions).

Figure 4 shows the rectal volume changes in the 5 patients analyzed from each week based on the kvCBCT scans contoured by the same radiation oncologist. Week 0 represents the rectal volume from the treatment planning kvCT scan. As seen in Fig. 4 below, there is a large variation in the rectal volume over the 9‐week period.

**Figure 5:**
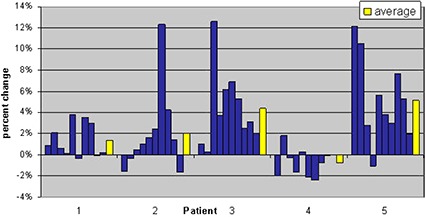
Change in percentage of rectal volume receiving 1.8 Gy relative to the treatment plan value listed by patient. Positive values indicate an increase in the volume of the rectum receiving 1.8 Gy; negative values indicate a decrease in rectal volume at that dose. Each column represents data from one selected daily cone‐beam scan per consecutive week of treatment. The average over these values for the course of treatment is shown by the yellow bars.

Radiation Therapy Oncology Group (RTOG) 0126 criteria of volume of rectum receiving 75 Gy (V 75 Gy) was chosen to track rectal doses from kvCBCT scans. This is equivalent to the percentage of rectal volume receiving the daily fraction dose of 180 cGy as compared to the rectal volume from the treatment planning kvCT scan. This is illustrated in Fig. 5. Due to the changes in rectal filling on the day of treatment, the maximum variation in rectal volume receiving the percentage of prescribed dose was as high as 12% (patient 3, Week 3).

**Figure 6:**
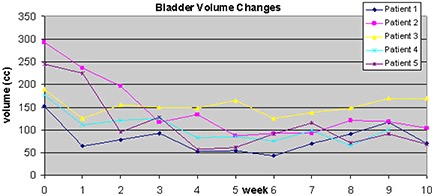
Change in bladder volume over the course of treatment (42 fractions) for all 5 patients.

**Figure 7:**
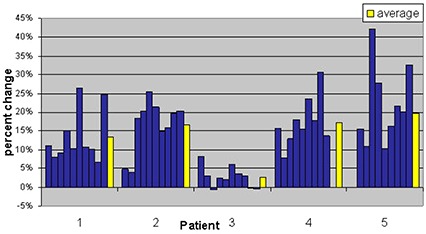
Change in percentage of bladder volume receiving 1.7 Gy relative to the treatment plan value listed by patient. Positive values indicate an increase in the volume of bladder receiving 1.7 Gy; negative values indicate a decrease in bladder volume at that dose. Each column represents data from one selected daily cone‐beam scan per consecutive week of treatment. The average for these values over the course of treatment is shown in yellow.

Figure 6 shows the bladder volume changes for the 5 patients treated using the Elekta Synergy system. There was a large variation in bladder volume, especially for patient 2, when compared against bladder volumes from the kvCT. In this study sample, the bladder volumes seem to decrease during treatment when compared to kvCT volumes. Radiation Therapy Oncology Group (RTOG) 0126 criteria of volume of bladder receiving 70 Gy (V 70 Gy) was chosen to track bladder doses from kvCBCT scans. This is equivalent to the percentage of bladder volume receiving the daily fraction dose of 170 cGy as compared to the bladder volume from kvCT scan. The results are reported in Fig. 7. Due to the changes in bladder filling on the day of treatment, the maximum variation in bladder volume receiving the percentage of prescribed dose was as high as 40% (patient 5, Week 3).

**Figure 8:**
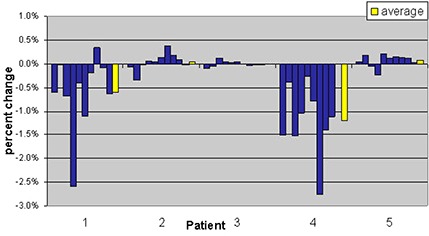
Change in mean target dose relative to the plan value for each patient. Each column represents data from one selected daily cone‐beam scan per consecutive week of treatment. The average for these values over the course of treatment is shown in yellow.

Finally, the changes in prostate target dose, based on recomputation of dose using the changes in the target volume as outlined in the kvCBCT images, were evaluated. The target dose change compared to planning dose is minimal as would be expected from positioning with daily image guidance. This is outlined in Fig. 8.

### C. Soft tissue contrast comparison of kvCT, kvCBCT, and MVCT scans

As expected, the kvCT images provided the best contrast resolution, while the MVCT displayed the poorest. The quantitative values for the 3 imaging modalities are listed in Table 1 (below).

**Table 1:** Quantitative values for contrast resolution in the 3 imaging modalities of MVCT, kvCBCT, and kvCT.

*Imaging Modality*	*3D Low‐contrast Visibility*	*3D Image Uniformity (%*)	*3D Spatial Resolution (line pairs*)
MVCT	3.19	9.3	4
kvCBCT	1.73	0.9	7
kvCT	0.11	0.044	7

### D. MVCT with tomotherapy

Of the 5 patients analyzed using an Adaptive Tomotherapy plan, 3 showed minimal differences between planned and delivered dose in terms of cumulative DVH. Instead of reporting cumulative doses received by each organ, the data were analyzed in terms of cumulative DVH as reported by the Planned Adaptive software. Three different scenarios arising from the 5 patient cases analyzed were picked to discuss adaptive radiotherapy strategies. A 10% difference between planned and delivered mean dose was used as the threshold for target and critical structures in deciding whether or not to reoptimize a given plan.

#### D.1 Scenario I ‐ Good agreement between planned and delivered dose (less than 5% difference between planned and delivered mean doses)

**Figure 9:**
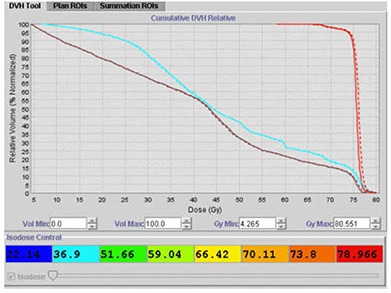
Good agreement between planned and delivered mean doses using Planned Adaptive software: (dashed line) summation dose; (solid line) planned dose; (cyan line) bladder; (brown line) rectum; (red line) prostate.

Figure 9 displays a scenario where there is good agreement between planned and delivered mean doses after manually contouring on 42 study sets to account for volume changes in the bladder, rectum, and prostate. The delivered summation dose for the prostate is slightly more than the planned dose. Overall, based on the Adaptive Tomotherapy plan, the actual delivered dose to the patient is in close agreement with the planned dose. In such a scenario, a new treatment plan with the merged studyset is not required.

#### D.2 Scenario II ‐ Minimal differences between planned and delivered dose (less than 10% difference between planned and delivered mean doses)

**Figure 10:**
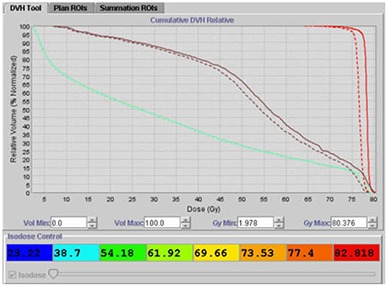
Minimal differences between planned and delivered mean doses using Planned Adaptive software: (dashed line) cumulative DVH; (solid line) planned DVH; (cyan line) bladder; (brown line) rectum; (red line) prostate.

For this patient, the cumulative DVH derived from the summation of verification doses is given below in Fig. 10. As shown in Fig. 10, the cumulative rectal DVH (dashed line) is less than the planned DVH for the rectum. The cumulative prostate DVH is less than the planned DVH for the prostate, with the prostate receiving slight underdosage even though the prescription dose is still covered by the 95% isodose line. Even though the planned and delivered mean doses differ slightly (less than 10% threshold limit), a plan modification using the merged study set would not be considered necessary in this scenario.

#### D.3 Scenario III ‐ Delivered dose NOT in agreement with planned dose (greater than 10% difference between planned and delivered mean doses)

**Figure 11:**
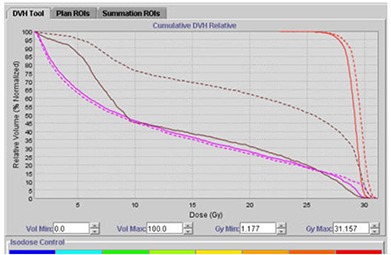
Large differences between planned and delivered doses using Planned Adaptive software: (dashed line) cumulative DVH; (solid line) planned DVH; (pink line) bladder; (brown line) rectum; (red line) prostate.

Figure 11 shows a patient for whom a Tomotherapy boost of 28.8 Gy over 16 fractions had been prescribed to be delivered to the prostate. In this patient, there was a large variation in dose delivered to the rectum when compared with the planned dose. The large variation was a result of the patient having a distended rectum during planning, which caused the volume of rectum irradiated during actual treatment delivery to be smaller in most fractions. A 10% difference between planned and delivered mean doses based on the mean dose statistics from the cumulative DVH was used as our threshold limit in deciding whether or not to reoptimize the plan based on the actual dose delivered. For this patient, after reviewing the cumulative DVH, the original plan was modified off‐line by choosing a different optimization scheme to account for the volume changes in the rectum from daily MVCT scans.

**Figure 12:**
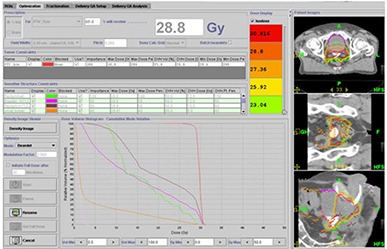
Reoptimized plan from the adaptive information whereby planned and delivered doses are now in agreement.

The resulting adapted plan is given below in Fig. 12, displaying that the planned and delivered doses to target, bladder and rectum are now in close agreement.

## IV. DISCUSSION

Positional variation of prostate gland in the treatment of prostate cancer has been extensively studied and various Image Guided Technologies, which can potentially correct for these variations, have also been reported.^(^ ^21^ ^–^ ^34^ ^)^

Several studies recently in the literature^(^ ^35^ ^–^ ^40^ ^)^ have shown that dose escalation is necessary and leads to an improved clinical outcome in the treatment of prostate cancer. Dose escalation, however, leads to increased dose to the critical structures, namely bladder and rectum, even with the IMRT treatment modality. There have also been studies that have demonstrated the efficacy of hypofractionated treatments for prostate cancer^(^ ^41^ ^–^ ^43^ ^)^ given the low α/β^(^ ^44^ ^–^ ^47^ ^)^ value suggested for prostate cancer. In this scenario, the precision and accuracy of the dose delivered to the target and critical structures takes on a greater significance. The evaluation of actual dose delivered to the prostate, bladder, and rectum based on the anatomy of the day may become a clinical necessity for these treatments.

Our study involving kvCBCT with the Elekta Synergy system clearly demonstrates that, in the absence of any special protocol that involves bowel preparation, daily soft tissue imaging with the kvCBCT scans show large variations in delivered dose to bladder and rectum with the confirmation that the dose delivered to the prostate is satisfactory. Thus, while clearly IGRT with daily soft tissue imaging improves the accuracy of dose delivered to prostate, it also has the potential to document and monitor changes in anatomy and dose to the critical structures.

**Figure 13:**
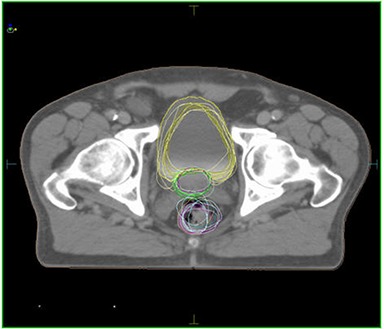
kvCT superimposed with kvCBCT contours showing variation in bladder and rectal volumes for a patient over a 9‐week period.

The changes in bladder and rectal volume were random in nature and the clinical impact of such variations cannot be well understood unless we quantify the changes and sum the doses from one CT scan to the other. The variations of bladder and rectal volumes from weekly kvCBCT scans are displayed on the treatment planning CT scan in Fig. 13. Currently, there are no commercial treatment planning systems that have the ability to carry out such an analysis in an automated manner. Consequently, even in this study which takes into account only a weekly kvCBCT scan for each patient, the time required to do a dosimetric analysis of this nature is not practical in a busy clinical setting. Working with the radiation oncologist, the physicists in this study spent approximately 3 hours per patient to contour bladder and rectum on each kvCBCT studyset.

Various strategies have been suggested in the literature for off‐line adaptive radiotherapy using kvCBCT scans.^(^ ^48^ ^–^ ^53^ ^)^ Most involve the creation of a modified target and rectum based on the evaluation of daily kvCBCT scans from the first few fractions, and a modified treatment plan created for the rest of the treatment course based on these structures.

We have shown that the maximum variation in rectum and bladder volumes in our kvCBCT study receiving the percentage of prescribed dose was 12% and 40%, respectively. These large variations are clinically significant and demonstrate the need for plan adaptation. Clearly, the challenge is to create cumulative DVH information to interpret the volume changes occurring during IGRT.

This was our motivation to perform the study with the Tomotherapy system using the Planned Adaptive software tool. As stated before, five patient plans were evaluated with this adaptive planning method to determine whether treatment plan improvements could be dosimetrically significant and to distinguish between clinically significant and clinically insignificant anatomy changes. The cumulative DVH information from the merged MVCT‐kvCT images gave us replanning options in case a significant discrepancy (10% or greater) existed between planned and delivered mean doses.

Deformation of organs is a complicated process if organ wall changes are to be quantified. The deformation of the organ wall was not included in our analysis for bladder and rectum using the MVCT images. The bladder and rectum were assumed to be “filled” organs during recontouring on the MVCT and kvCBCT studysets. The soft tissue contrast was found to be insufficient for organ wall delineation. A sample MVCT image with and without the original kvCT contours are given below in Fig. 14(a) and 14(b) to illustrate this point.

**Figure 14(a):**
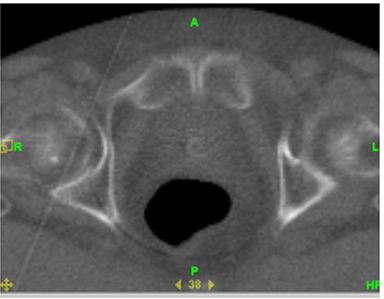
MVCT scan of patient illustrating lack of sufficient image contrast for rectal wall delineation.

**Figure 14(b):**
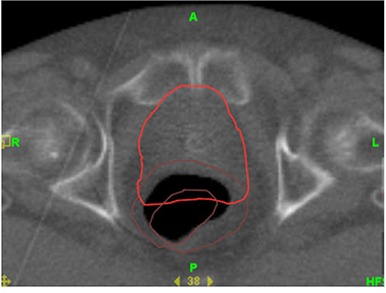
Same MVCT scan with rectal volumes from kvCT and MVCT drawn but rectal wall cannot be visualized for delineation.

The replanning options can be divided into the two broad strategies of off‐line and on‐line options. The off‐line approach is the most practically feasible approach to implement today and a practical implementation strategy using the Planned Adaptive software on the tomotherapy system is provided here. The off‐line approach requires cumulating all of the actual delivered doses by accounting for the daily volume changes of prostate, bladder, and rectum. Therefore, in principle, a cumulative DVH can be created at the end of each week to evaluate any potential changes in delivered dose as compared to planned dose delivery. If a significant discrepancy occurs (greater than the 10% mean dose difference threshold) as shown in Scenario III above, a reoptimization of the plan will be done to account for these changes. A final plan will then be created, which results in a DVH that closely matches or has a superior dose distribution to the original planned distribution based on the feedback from the changed anatomy.

In cases where there are only minimal differences in the cumulative DVH at the end of each week of treatment, the original plan will continue to be used for patient treatment and a cumulative DVH will be created at the end of patient treatment demonstrating that the planned and actual dose are in agreement. The cumulative DVH for these cases will serve as a clinical quality assurance tool to document that the actual delivered doses were in agreement with the planned dose.

The main drawback of performing the planned adaptive plans is that they are extremely time‐consuming because all the contours have to be manually drawn as in our kvCBCT study. An average of 12 hours per patient was spent to contour an entire 42 fraction MVCT studyset. The other major drawback is that, although the summation dose is computed in the planned adaptive software, this only evaluates the summation dose for one MVCT at a time and does not include a deformable registration model, which can potentially follow the doses delivered to the voxels creating an overall dose pattern. We are currently actively pursuing deformable registration tools with MVCT to create such models.

The on‐line adaptive therapy process accounts for the deformation of the prostate, bladder, and rectum using deformable registration tools based on the anatomy whereby DVH is created and compared to the planned DVH while the patient is on the table. Thus any changes to the plan or positioning of the patient is done not just by image registration data but after on‐line evaluation of dose. This process can only be implemented if there are automatic software tools which evaluate the deformation of the prostate, bladder, and rectum in real time and feed the information to the optimization engine such that DVH can be generated in real time while the patient is still on table.

## V. CONCLUSIONS

Our study involving both kvCBCT and MVCT image guidance has shown the ability to track actual doses delivered to prostate, bladder, and rectum based on anatomy of the day. Due to the large variation in CT number on the kvCBCT images with the Elekta Synergy system, we conclude that direct dose computation on these images is not feasible. We have also quantitatively evaluated the low contrast resolution, spatial resolution, and image uniformity of three imaging modalities (kvCT, kvCBCT, MVCT) using Catphan600 phantom and have found, as expected, that the kvCT and kvCBCT images have better contrast resolution than the MVCT images.

Using the Planned Adaptive software on the tomotherapy system, our study has demonstrated the ability to sum doses from multiple fractions on the merged kvCT/MVCT studyset in order to construct and evaluate a cumulative DVH. We have demonstrated the need for a clinical process where, using the adapted plan, an adjustment to treatment plan optimization may be performed whereby actual delivered doses are in agreement with the planned dose based on the information gained from daily MVCT scans. To take this investigation further, one has to develop deformable registration tools that can be used to calculate cumulative doses to organs at risk and target volumes, thereby providing a valuable tool for evaluating adapted plans. It is our belief that such evaluations will eventually pave the way for a dose‐guided radiotherapy paradigm in the treatment of localized prostate cancer.

## ACKNOWLEDGEMENTS

The authors wish to thank the anonymous Reviewer A whose constructive suggestions helped to substantially improve the manuscript.
